# Supplementary material for: Association between Problematic Internet and Mobile Phone Use, autistic traits, and psychological distress among adults: A cross-sectional survey
Source: PLOS Ment Health. 2026 Jun 2;3(6):e0000524. doi: 10.1371/journal.pmen.0000524 (PMC13229353; doi:10.1371/journal.pmen.0000524)
Supplement: S1 Table — (DOCX) [file pmen.0000524.s001.docx]

**Association Between Problematic Internet and Mobile Phone Use, Autistic Traits, and Psychological Distress Among Adults: A Cross-Sectional Survey**

Matilda Floris, Claudio Gentili

**S1 Table.** Sociodemographic and Internet and mobile phone use variables of the participants who completed the IAT (n= 230) compared with those who did not complete IAT (n= 214).

|  | **Samples** | |  |  |
| --- | --- | --- | --- | --- |
| **Variables** | **IAT**  n = 230 | **No IAT**  n = 214 | **Statistical Test** | ***p-value*** |
| **Gender** |  |  | Fisher’s Exact Test | 0.513 |
| Female | 177 (82%) | 165 (77%) |  |  |
| Male | 36 (17%) | 45 (21%) |  |  |
| Non binary | 1 (0.5%) | 1 (0.5%) |  |  |
| Prefer to not answer | 1 (0.5%) | 3 (1.5%) |  |  |
| **Age** |  |  | W = 25005 | 0.770 |
| Mean (SD) | 39 (14) | 39 (14) |  |  |
| Median | 38 | 37 |  |  |
| Min - Max | 18 - 75 | 18 - 65 |  |  |
| **Education level** |  |  | χ^2^ (4, N = 444) = 5.38) | 0.250 |
| High school | 80 (37%) | 90 (42%) |  |  |
| Master's degree | 49 (23%) | 49 (23%) |  |  |
| Bachelor's degree | 47 (22%) | 42 (20%) |  |  |
| Post-lauream | 31 (14%) | 19 (9%) |  |  |
| Middle school | 8 (4%) | 14 (6%) |  |  |
| **Citizenship** |  |  | Fisher’s Exact Test | 0.498 |
| Italian | 213 (99%) | 214 (100%) |  |  |
| European | 2 (1%) | 0 (0%) |  |  |
| **Origin** |  |  | Fisher’s Exact Test | 0.148 |
| Islands | 131 (61%) | 139 (65%) |  |  |
| North Italy | 50 (23%) | 55 (26%) |  |  |
| Central Italy | 15 (7.0%) | 11 (5%) |  |  |
| South Italy | 13 (6.0%) | 9 (4%) |  |  |
| Europe | 5 (2.5%) | 0 (0%) |  |  |
| Extra-Europe | 1 (0.5%) | 0 (0%) |  |  |
| **Marital status** |  |  | Fisher’s Exact Test | 0.743 |
| Relationship | 83 (39%) | 90 (42%) |  |  |
| Married | 66 (31%) | 72 (34%) |  |  |
| Single | 52 (24%) | 43 (20%) |  |  |
| Divorced | 6 (2.5%) | 3 (1.5%) |  |  |
| Separated | 6 (2.5%) | 5 (2%) |  |  |
| Widowed | 2 (1%) | 1 (0.5%) |  |  |
| **Housing** |  |  | Fisher’s Exact Test | 0.407 |
| With partner | 98 (46%) | 109 (51%) |  |  |
| With parents | 43 (20%) | 33 (15%) |  |  |
| Alone | 32 (15%) | 35 (16%) |  |  |
| With roommates | 24 (11%) | 27 (13%) |  |  |
| Other | 17 (7.5%) | 10 (5%) |  |  |
| University residence | 1 (0.5%) | 0 (0%) |  |  |
| **Occupation** |  |  | Fisher’s Exact Test | 0.678 |
| Worker | 131 (61%) | 132 (62%) |  |  |
| Student | 43 (20%) | 38 (18%) |  |  |
| Student and worker | 21 (10%) | 27 (13%) |  |  |
| Retired | 12 (5.5%) | 7 (3%) |  |  |
| Unemployed | 8 (3.5%) | 8 (3.5%) |  |  |
| Unable to work | 0 (0%) | 1 (0.5%) |  |  |
| **Mobile Phone** |  |  | χ^2^ (3, N = 444) = 0.67) | 0.880 |
| 2-5 hours | 122 (57%) | 124 (58%) |  |  |
| 5-8 hours | 30 (14%) | 24 (11%) |  |  |
| 8+ hours | 7 (3%) | 7 (3%) |  |  |
| Less than 2 hours | 56 (26%) | 57 (27%) |  |  |
| **Social network** |  |  | Fisher’s Exact Test | 0.923 |
| 2 hours | 131 (61%) | 134 (63%) |  |  |
| 5 hours | 5 (2.3%) | 4 (2.2%) |  |  |
| 7 hours | 21 (9.7%) | 16 (7.6%) |  |  |
| 7+ hours | 55 (26%) | 54 (25%) |  |  |
| Never | 3 (1%) | 4 (2.2%) |  |  |
| **Mobile Phone Use** |  |  | Fisher’s Exact Test | 0.519 |
| Communication | 113 (53%) | 98 (46%) |  |  |
| Games | 6 (2.5%) | 7 (3%) |  |  |
| Internet navigation | 0 (0%) | 3 (1.5%) |  |  |
| Other | 21 (9.5%) | 21 (9.5%) |  |  |
| Shopping | 7 (3%) | 9 (5%) |  |  |
| Social network | 68 (32%) | 74 (35%) |  |  |
| **UADI-2** |  |  | W = 25455 | 0.531 |
| Mean (SD) | 57 (15) | 56 (16) |  |  |
| Median | 56 | 56 |  |  |
| Min - Max | 27 - 99 | 25 - 106 |  |  |

UADI-2: Internet Use-Abuse and Addiction (Questionario Uso-Abuso e Dipendenza da Internet 2)
