# Supplementary material for: Association between Problematic Internet and Mobile Phone Use, autistic traits, and psychological distress among adults: A cross-sectional survey
Source: PLOS Ment Health. 2026 Jun 2;3(6):e0000524. doi: 10.1371/journal.pmen.0000524 (PMC13229353; doi:10.1371/journal.pmen.0000524)
Supplement: S2 Table — (DOCX) [file pmen.0000524.s002.docx]

**Association Between Problematic Internet and Mobile Phone Use, Autistic Traits, and Psychological Distress Among Adults: A Cross-Sectional Survey**

Matilda Floris, Claudio Gentili

**S2 Table. Main analyses excluding the Imagination subscale of the AQ.**

Items of Imagination subscale were removed and the AQ total score was recalculated. The new AQ total score showed a global Cronbach’s α of .82, as the original analysis, indicating a good internal consistency.

We re-run Spearman’s correlations between corrected AQ score (without Imagination subscale) and each dependent variable (UADI-2, MPPUS, ASSIST-alcohol and ASSIST-tobacco) across age groups. The table below summarized both the new correlations based on AQ corrected score and the original correlations reported in Table 3 of the manuscript. Although minor numerical differences emerged, the overall pattern of results is unchanged.

| Age groups | Variables | Rho (original) | *p-value* (original) | Rho (without Imagination subscale) | *p-value* (without Imagination subscale) | n |
| --- | --- | --- | --- | --- | --- | --- |
| **18–24** | ASSIST - Alcohol | -0.17 | 0.073 | -0.14 | 0.148 | 114 |
| **18–24** | ASSIST – Tobacco | -0.19 | 0.049 | -0.19 | 0.046 | 114 |
| **18–24** | MPPUS | 0.41 | < .001 | 0.39 | < .001 | 114 |
| **18–24** | UADI2 | 0.46 | < .001 | 0.48 | < .001 | 114 |
| **25–36** | ASSIST - Alcohol | -0.04 | 0.691 | -0.05 | 0.583 | 107 |
| **25–36** | ASSIST – Tobacco | -0.06 | 0.555 | -0.03 | 0.772 | 107 |
| **25–36** | MPPUS | 0.42 | < .001 | 0.46 | < .001 | 107 |
| **25–36** | UADI2 | 0.50 | < .001 | 0.53 | < .001 | 107 |
| **37–49** | ASSIST - Alcohol | 0.06 | 0.515 | 0.07 | 0.446 | 106 |
| **37–49** | ASSIST – Tobacco | -0.09 | 0.369 | -0.05 | 0.576 | 106 |
| **37–49** | MPPUS | 0.24 | 0.015 | 0.26 | 0.007 | 106 |
| **37–49** | UADI2 | 0.30 | 0.002 | 0.33 | < .001 | 106 |
| **50–65** | ASSIST - Alcohol | 0.14 | 0.194 | 0.13 | 0.213 | 93 |
| **50–65** | ASSIST – Tobacco | 0.07 | 0.517 | 0.07 | 0.487 | 93 |
| **50–65** | MPPUS | 0.12 | 0.238 | 0.19 | 0.071 | 93 |
| **50–65** | UADI2 | 0.22 | 0.033 | 0.29 | 0.006 | 93 |

MANCOVA were not performed using the AQ corrected score, as this analysis required categorical grouping (AQ traits). We cannot compute AQ traits without the Imagination subscale, as the established cut-off (≥ 26) does not retain the same validity. Thus, multivariate multiple regression (MMR) was re-run treating the correct AQ score as a continuous variable. The results align with those of the original analysis:

- PIU (UADI-2) was significantly predicted by both AQ (β = 0.69, SE = 0.08, t(417) = 8.41, p < .001) and K10 (β = 0.72, SE = 0.11, t(417) = 6.16, p < .001), as in the original analysis
- PMPU (MPPUS) was significantly predicted by both AQ (β = 0.42, SE = 0.11, t(417) = 3.62, p < .001) and K10 (β = 0.83, SE = 0.09, t(417) = 9.18, p < .001), as in the original analysis
- Alcohol use (ASSIST) was significantly predicted by only K10 (β = 0.16, SE = 0.03, t(417) = 4.38, p < .001), as in the original analysis
- Tobacco use (ASSIST) was significantly predicted by only K10 (β = 0.36, SE = 0.06, t(417) = 6.11, p < .001), as in the original analysis

These results support the robustness of the main findings, indicating that the poor internal consistency of the AQ Imaginal subscale did not substantially affect the results.
