# Supplementary material for: Association between Problematic Internet and Mobile Phone Use, autistic traits, and psychological distress among adults: A cross-sectional survey
Source: PLOS Ment Health. 2026 Jun 2;3(6):e0000524. doi: 10.1371/journal.pmen.0000524 (PMC13229353; doi:10.1371/journal.pmen.0000524)
Supplement: S3 Table — (DOCX) [file pmen.0000524.s003.docx]

**Association Between Problematic Internet and Mobile Phone Use, Autistic Traits, and Psychological Distress Among Adults: A Cross-Sectional Survey**

Matilda Floris, Claudio Gentili

**S3 Table. Description of sociodemographic variables**

Gender, age groups, education level, geographic origin, marital status, housing condition, occupation, economic level, presence of chronic disease, presence of psychological disorders, issues with justice ever in life, traumatic event, time spent on social network, time spent on smartphone, principal activity of phone daytime were used as factors in the models. Each variable was assessed with self-report questions of the sociodemographic questionnaire, and was used as categorical variables. Details of each variable are provided in the table below.

| **Variables** | **Question** | **Details of levels** |
| --- | --- | --- |
| Gender | *Gender:* | 1. Male 2. Female 3. Non-binary 4. Prefer to not answer |
| Education level | *Last qualification obtained:* | 1. Elementary school 2. Middle-school 3. High-school 4. Bachelor’s degree master’s degree 5. Post-lauream specialization (eg. Phd) |
| Citizenship | *Citizenship:* | 1. Italian 2. European 3. Extra-European |
| Origin | *Place of birth:* | 1. North Italy 2. Central Italy 3. South Italy 4. Islands (Italy) 5. Europe 6. Extra-Europe |
| Marital status | *Marital status:* | 1. Single 2. In a relationship 3. Married 4. Separated 5. Divorced 6. Widower/widow |
| Housing | *Most of the time, you live:*  *If other, we kindly ask to specify.* | 1. Alone 2. With my parents 3. With my partner 4. With roommates 5. In university student residence 6. Other* |
| Occupation | *Occupation:* | 1. Student 2. Worker 3. Student and worker 4. Unemployed 5. Retire 6. Disabled |
| Economic level | *Personal or familial net income per year:* | 1. Up to 10k 2. 10k – 15k 3. 15k – 20k 4. 20k – 30k 5. 30 – 50k 6. More than 50k 7. I prefer to not answer |
| Chronic disease | *Do you suffer from a chronic medical condition?* | 1. Yes* 2. No |
| Psychological disorder | *Do you suffer or have you ever suffered from a psychological disorder?*  *If yes, we kindly ask to specify.* | 1. Yes* 2. No |
| Traumatic event | *In your life have you ever had at least one experience that you consider traumatic or particularly negative?* | 1. Yes* 2. No 3. I prefer to not answer |
| Issue with justice | *Have you ever had problems with the justice system?* | 1. No, of any kind 2. Yes, civil issues 3. Yes, penal issues 4. Yes, I was detained |
| Familiarity | *Among his first-degree family members has anyone ever suffered from a form of addiction?* | 1. No, of any kind 2. Yes, from a substance addiction 3. Yes, from gambling |
| Social network | *On a typical day, how many hours do you spend using social networks (Facebook Instagram Tik-Tok Youtube)* | 1. Up to 2 hours 2. Up to 5 hours 3. Up to 7 hours 4. More than 7 hours 5. I never use social-networks |
| Mobile Phone | *On a typical day how many hours a day do you use your smartphone?* | 1. Less than 2 hours 2. 2-5 hours per day 3. 5-8 hours per day 4. More than 8 hours |
| Type of mobile phone use | *What activity do you mainly and most of the time do with your smartphone?*  *If other, we kindly ask to specify.* | 1. Communication (calls, messages, emails) 2. Social network 3. Games 4. Shopping 5. Web surfing 6. Other* |

*Participants might provide additional details – e.g., specifying their psychological diagnosis – in a text box attached to the item, but it was not mandatory.
