# Supplementary material for: Association between Problematic Internet and Mobile Phone Use, autistic traits, and psychological distress among adults: A cross-sectional survey
Source: PLOS Ment Health. 2026 Jun 2;3(6):e0000524. doi: 10.1371/journal.pmen.0000524 (PMC13229353; doi:10.1371/journal.pmen.0000524)
Supplement: S4 Table — (DOCX) [file pmen.0000524.s004.docx]

**Association Between Problematic Internet and Mobile Phone Use, Autistic Traits, and Psychological Distress Among Adults: A Cross-Sectional Survey**

Matilda Floris, Claudio Gentili

**S4 Table. Descriptive analysis of subsample of males and females (n= 414) by gender.**

|  | **Gender** | |  |  |
| --- | --- | --- | --- | --- |
| **Variables** | **Females**  n = 334 | **Males**  n = 80 |  | ***p-value*** |
| **AQ** |  |  | W = 10291 | 0.001 |
| Mean (SD) | 16.30 (6.90) | 18.93 (6.69) |  |  |
| Median | 15 | 18.50 |  |  |
| Min - Max | 2 – 42 | 6 - 33 |  |  |
| **K10** |  |  | W= 13938 | 0.547 |
| Mean (SD) | 22.51 (8.43) | 21.51 (7.31) |  |  |
| Median | 20 | 20 |  |  |
| Min - Max | 10 - 48 | 10 - 48 |  |  |
| **UADI-2** |  |  | W= 10377 | 0.001 |
| Mean (SD) | 55.96 (15.30) | 61.90 (16.21) |  |  |
| Median | 55 | 63 |  |  |
| Min - Max | 25 - 106 | 25 - 99 |  |  |
| **MPPUS** |  |  | W = 12914 | 0.643 |
| Mean (SD) | 53.08 (15.90) | 54.40 (16.54) |  |  |
| Median | 52 | 53 |  |  |
| Min - Max | 24 - 96 | 24 - 93 |  |  |
| **ASSIST - Tabacco** |  |  | W = 13133 | 0.799 |
| Mean (SD) | 6.94 (9.29) | 6.70 (9.25) |  |  |
| Median | 0 | 2 |  |  |
| Min - Max | 0- 36 | 0 - 34 |  |  |
| **ASSIST - Alcohol** |  |  | W = 10198 | <0.001 |
| Mean (SD) | 5.34 (5.24) | 7.75 (6.57) |  |  |
| Median | 4 | 6 |  |  |
| Min - Max | 0 - 24 | 0 - 27 |  |  |

AQ: Autistic Spectrum Quotient; K10: Kessler Psychological Distress Scale; UADI-2: Uso-Abuso e Dipendenza da Internet 2; MPPUS: Mobile Phone Problem Usage Scale; ASSIST: The Alcohol, Smoking and Substance Involvement Screening Test
