# Supplementary material for: Association between Problematic Internet and Mobile Phone Use, autistic traits, and psychological distress among adults: A cross-sectional survey
Source: PLOS Ment Health. 2026 Jun 2;3(6):e0000524. doi: 10.1371/journal.pmen.0000524 (PMC13229353; doi:10.1371/journal.pmen.0000524)
Supplement: S5 Table — (DOCX) [file pmen.0000524.s005.docx]

**Association Between Problematic Internet and Mobile Phone Use, Autistic Traits, and Psychological Distress Among Adults: A Cross-Sectional Survey**

Matilda Floris, Claudio Gentili

**S5 Table. MANOVA and ANCOVAs of subsample (n= 414, males and females).**

**5a.** MANCOVA: *(ASSIST-Alcohol, ASSIST-Tobacco, UADI-2, MPPUS, AQ, K10) ~ Gender*

| **Variable** | **Test** | **Value** | **F (6, 407)** | ***p value*** |
| --- | --- | --- | --- | --- |
| Gender | Pillai’s Trace | .104 | 7.94 | < .001 |

**5b**. ANOVAs

| **Variables** | **F (df1, df2)** | ***p-value*** | **η²** | **95% CI [Lower, Upper]** |
| --- | --- | --- | --- | --- |
| AQ | 9.42 (1,412) | .002 | .02 | [.01, 1.00] |
| K10 | 0.94 (1,412) | .331 | .002 | [.00, 1.00] |
| UADI-2 | 9.28 (1,412) | .002 | .02 | [.00, 1.00] |
| MPPUS | 0.43 (1,412) | .51 | .001 | [.00, 1.00] |
| ASSIST - Alcohol | 12.37 (1,412) | < .001 | .03 | [.01, 1.00] |
| ASSIST - Tobacco | 0.04 (1,412) | .836 | .001 | [.00, 1.00] |

AQ: Autistic Spectrum Quotient; K10: Kessler Psychological Distress Scale; UADI-2: Uso-Abuso e Dipendenza da Internet 2; MPPUS: Mobile Phone Problem Usage Scale; ASSIST: The Alcohol, Smoking and Substance Involvement Screening Test
