# Supplementary material for: Association between Problematic Internet and Mobile Phone Use, autistic traits, and psychological distress among adults: A cross-sectional survey
Source: PLOS Ment Health. 2026 Jun 2;3(6):e0000524. doi: 10.1371/journal.pmen.0000524 (PMC13229353; doi:10.1371/journal.pmen.0000524)
Supplement: S6 Table — (DOCX) [file pmen.0000524.s006.docx]

**Association Between Problematic Internet and Mobile Phone Use, Autistic Traits, and Psychological Distress Among Adults: A Cross-Sectional Survey**

Matilda Floris, Claudio Gentili

**S6 Table. Other sociodemographic variables among included participants (n= 420).**

|  | **Age groups** | | | | |  |  |
| --- | --- | --- | --- | --- | --- | --- | --- |
| **Variables** | **Overall**  N = 420 | **18–24**  n = 114 | **25–36**  n = 107 | **37–49**  n = 106 | **50–65**  n = 93 | **Statistical test** | ***p-value*** |
| **Economic level** |  |  |  |  |  | Fisher’s Exact Test | <0.001 |
| 10.000 € | 60 (14%) | 26 (23%) | 22 (21%) | 8 (7.5%) | 4 (4.3%) |  |  |
| 10.001 - 15.000 € | 49 (12%) | 13 (11%) | 19 (18%) | 13 (12%) | 4 (4.3%) |  |  |
| 15.001 - 20.000 € | 62 (15%) | 10 (8.8%) | 19 (18%) | 20 (19%) | 13 (14%) |  |  |
| 20.001 - 30.000 € | 118 (28%) | 19 (17%) | 22 (21%) | 37 (35%) | 40 (43%) |  |  |
| 30.001 - 50.000 € | 52 (12%) | 13 (11%) | 11 (10%) | 11 (10%) | 17 (18%) |  |  |
| < 50.000€ | 14 (4%) | 3 (2.2%) | 3 (3%) | 3 (2.5%) | 5 (5.4%) |  |  |
| Prefer to not answer | 64 (15%) | 30 (26%) | 11 (10%) | 14 (13%) | 9 (10%) |  |  |
| **Psychological diagnosis (yes)** | 53 (13%) | 20 (18%) | 18 (17%) | 7 (6.6%) | 8 (8.8%) | χ^2^ (3, N = 420) = 8.87) | .031 |
| **Issues with justice** |  |  |  |  |  | Fisher’s Exact Test | 0.989 |
| No | 409 (98%) | 112 (98%) | 104 (97%) | 103 (97%) | 90 (99%) |  |  |
| Yes (civil) | 6 (1.3%) | 1 (1%) | 2 (2%) | 2 (2%) | 1 (2%) |  |  |
| Imprisoned | 3 (0.7%) | 1 (1%) | 1 (1%) | 1 (1%) | 0 (0%) |  |  |
| **Familiarity** |  |  |  |  |  | Fisher’s Exact Test | 0.416 |
| No | 343 (82%) | 90 (79%) | 91 (85%) | 84 (79%) | 78 (86%) |  |  |
| Yes, substance addiction | 66 (16%) | 19 (17%) | 15 (14%) | 21 (20%) | 11 (12%) |  |  |
| Yes, behavioural addiction | 9 (3%) | 5 (4%) | 1 (1%) | 1 (1%) | 2 (2%) |  |  |
| **Trauma** |  |  |  |  |  | χ^2^ (6, N= 420) = 3.22 | 0.770 |
| No | 186 (44%) | 49 (43%) | 48 (45%) | 50 (47%) | 39 (43%) |  |  |
| Yes | 155 (37%) | 44 (39%) | 44 (41%) | 34 (32%) | 33 (36%) |  |  |
| Prefer to not answer | 77 (18%) | 21 (18%) | 15 (14%) | 22 (21%) | 19 (21%) |  |  |
