# Supplementary material for: Association between Problematic Internet and Mobile Phone Use, autistic traits, and psychological distress among adults: A cross-sectional survey
Source: PLOS Ment Health. 2026 Jun 2;3(6):e0000524. doi: 10.1371/journal.pmen.0000524 (PMC13229353; doi:10.1371/journal.pmen.0000524)
Supplement: S7 Table — (DOCX) [file pmen.0000524.s007.docx]

**Association Between Problematic Internet and Mobile Phone Use, Autistic Traits, and Psychological Distress Among Adults: A Cross-Sectional Survey**

Matilda Floris, Claudio Gentili

**S7 Table. Details of psychological diagnosis.**

|  |  | **Autistic traits** | |
| --- | --- | --- | --- |
| **Psychological diagnoses** | **Overall**  N = 420 | **Low autistic traits**  n = 359 | **High autistic traits**  n = 61 |
| Anxiety disorders | 20 | 13 | 7 |
| Depression | 19 | 14 | 5 |
| Personality disorders | 2 | 1 | 1 |
| Obsessive-compulsive disorder | 4 | 3 | 1 |
| Dissociative Disorder | 1 | 1 | 0 |
| Post-traumatic stress disorder | 3 | 2 | 1 |
| Attention Deficit Hyperactivity Disorder | 3 | 2 | 1 |
| Learning disabilities | 4 | 4 | 0 |
| Autism spectrum disorder | 1 | 0 | 1 |
| Eating disorders | 2 | 0 | 2 |
