# Supplementary material for: Association between Problematic Internet and Mobile Phone Use, autistic traits, and psychological distress among adults: A cross-sectional survey
Source: PLOS Ment Health. 2026 Jun 2;3(6):e0000524. doi: 10.1371/journal.pmen.0000524 (PMC13229353; doi:10.1371/journal.pmen.0000524)
Supplement: S8 Table — (DOCX) [file pmen.0000524.s008.docx]

**Association Between Problematic Internet and Mobile Phone Use, Autistic Traits, and Psychological Distress Among Adults: A Cross-Sectional Survey**

Matilda Floris, Claudio Gentili

**S8 Table. Use of mobile phone and Internet among participants (n= 420) by age groups.**

|  | **Age groups** | | | | |  |  |
| --- | --- | --- | --- | --- | --- | --- | --- |
| **Variables** | **Overall**  N = 420 | **18–24**  n = 114 | **25–36**  n = 107 | **37–49**  n = 106 | **50–65**  n = 93 | **Statistical test** | ***p-value*** |
| **Mobile Phone** |  |  |  |  |  | Fisher’s Exact Test | <0.001 |
| 2-5 hours | 242 (58%) | 73 (64%) | 74 (69%) | 55 (52%) | 40 (44%) |  |  |
| 5-8 hours | 54 (13%) | 25 (22%) | 14 (13%) | 9 (8.5%) | 6 (7%) |  |  |
| 8+ hours | 14 (3%) | 7 (6.1%) | 4 (3%) | 3 (2.5%) | 0 (0%) |  |  |
| Less than 2 hours | 108 (26%) | 9 (7.9%) | 15 (14%) | 39 (37%) | 45 (49%) |  |  |
| **Social network** |  |  |  |  |  | Fisher’s Exact Test | <0.001 |
| 2 hours | 261 (62%) | 47 (41%) | 70 (65%) | 76 (72%) | 68 (75%) |  |  |
| 5 hours | 9 (2.2%) | 6 (6%) | 2 (2%) | 1 (1%) | 0 (0%) |  |  |
| 7 hours | 34 (8%) | 2 (2%) | 4 (4%) | 14 (13%) | 14 (15%) |  |  |
| 7+ hours | 107 (26%) | 54 (47%) | 30 (28%) | 14 (13%) | 9 (10%) |  |  |
| Never | 7 (1.7%) | 5 (4%) | 1 (1%) | 1 (1%) | 0 (0%) |  |  |
| **Mobile Phone Use** |  |  |  |  |  | Fisher’s Exact Test | <0.001 |
| Communication | 206 (49%) | 43 (38%) | 46 (43%) | 55 (52%) | 62 (68%) |  |  |
| Games | 12 (3%) | 7 (6%) | 2 (2%) | 3 (2.5%) | 0 (0%) |  |  |
| Internet navigation | 3 (1%) | 0 (0%) | 2 (2%) | 1 (1%) | 0 (0%) |  |  |
| Other | 40 (10%) | 6 (5%) | 6 (5.4%) | 19 (18%) | 9 (10%) |  |  |
| Shopping | 16 (3 %) | 1 (1%) | 3 (2.6%) | 3 (2.5%) | 9 (10%) |  |  |
| Social network | 141 (34%) | 57 (50%) | 48 (45%) | 25 (24%) | 11 (12%) |  |  |
