# Supplementary material for: Association between Problematic Internet and Mobile Phone Use, autistic traits, and psychological distress among adults: A cross-sectional survey
Source: PLOS Ment Health. 2026 Jun 2;3(6):e0000524. doi: 10.1371/journal.pmen.0000524 (PMC13229353; doi:10.1371/journal.pmen.0000524)
Supplement: S9 Table — (DOCX) [file pmen.0000524.s009.docx]

**Association Between Problematic Internet and Mobile Phone Use, Autistic Traits, and Psychological Distress Among Adults: A Cross-Sectional Survey**

Matilda Floris, Claudio Gentili

**S9 Table. Percentages and Chi-squared of substances use based on ASSIST scores (Used: ASSIST > 0; Not used: ASSIST = 0).**

|  | **Age groups** | | | | |  |  |
| --- | --- | --- | --- | --- | --- | --- | --- |
| **Variables** | **Overall**  N = 420 | **18–24**  n = 114 | **25–36**  n = 107 | **37–49**  n = 106 | **50–65**  n = 93 | **Statistical test** | ***p-value*** |
| **Alcohol** |  |  |  |  |  | χ^2^ (3, N= 420) = 15.24 | 0.002 |
| Not used | 90 (21%) | 18 (16%) | 15 (14%) | 25 (24%) | 32 (34%) |  |  |
| Used | 330 (79%) | 96 (84%) | 92 (86%) | 81 (76%) | 61 (66%) |  |  |
| **Tobacco** |  |  |  |  |  | χ^2^ (3, N= 420) = 22.81 | <0.001 |
| Not used | 216 (51%) | 42 (37%) | 48 (45%) | 67 (63%) | 59 (63%) |  |  |
| Used | 204 (49%) | 72 (63%) | 59 (55%) | 39 (37%) | 34 (37%) |  |  |
| **Cannabis** |  |  |  |  |  | χ^2^ (3, N= 420) = 34.50 | <0.001 |
| Not used | 325 (77%) | 68 (60%) | 83 (78%) | 88 (83%) | 86 (92%) |  |  |
| Used | 95 (23%) | 46 (40%) | 24 (22%) | 18 (17%) | 7 (8%) |  |  |
| **Cocaine** |  |  |  |  |  | Fisher’s Exact Test | 0.112 |
| Not used | 413 (98%) | 114 (100%) | 103 (96%) | 105 (99%) | 91 (98%) |  |  |
| Used | 7 (2%) | 0 (0%) | 4 (4%) | 1 (1%) | 2 (2%) |  |  |
| **Amphetamine** |  |  |  |  |  | Fisher’s Exact Test | 0.153 |
| Not used | 415 (99%) | 112 (98%) | 104 (97%) | 106 (100%) | 93 (100%) |  |  |
| Used | 5 (1%) | 2 (2%) | 3 (3%) | 0 (0%) | 0 (0%) |  |  |
| **Inhalants** |  |  |  |  |  | Fisher’s Exact Test | 0.595 |
| Not used | 418 (99.5%) | 113 (99%) | 107 (100%) | 106 (100%) | 92 (99%) |  |  |
| Used | 2 (0.5%) | 1 (1%) | 0 (0%) | 0 (0%) | 1 (1%) |  |  |
| **Sedatives** |  |  |  |  |  | χ^2^ (3, N= 420) = 33.54 | 0.314 |
| Not used | 397 (95%) | 111 (97%) | 101 (94%) | 100 (94%) | 85 (91%) |  |  |
| Used | 23 (5 %) | 3 (3%) | 6 (6%) | 6 (6%) | 8 (9%) |  |  |
| **Hallucinogens** |  |  |  |  |  | Fisher’s Exact Test | 0.016 |
| Not used | 414 (99%) | 109 (96%) | 107 (100%) | 105 (99%) | 93 (100%) |  |  |
| Used | 6 (1%) | 5 (4%) | 0 (0%) | 1 (1%) | 0 (0%) |  |  |
| **Opioids** |  |  |  |  |  | Fisher’s Exact Test | 1 |
| Not used | 419 (98.8%) | 113 (99%) | 107 (100%) | 106 (100%) | 93 (100%) |  |  |
| Used | 1 (0.2%) | 1 (1%) | 0 (0%) | 0 (0%) | 0 (0%) |  |  |

ASSIST: The Alcohol, Smoking and Substance Involvement Screening Test
