# Supplementary material for: Association between Problematic Internet and Mobile Phone Use, autistic traits, and psychological distress among adults: A cross-sectional survey
Source: PLOS Ment Health. 2026 Jun 2;3(6):e0000524. doi: 10.1371/journal.pmen.0000524 (PMC13229353; doi:10.1371/journal.pmen.0000524)
Supplement: S10 Table — (DOCX) [file pmen.0000524.s010.docx]

**Association Between Problematic Internet and Mobile Phone Use, Autistic Traits, and Psychological Distress Among Adults: A Cross-Sectional Survey**

Matilda Floris, Claudio Gentili

**S10 Table**. **Spearman correlation between AQ or K10 and dependent variables among all participants (n= 420).**

| Variable 1 | Variable 2 | rho | *p value* |
| --- | --- | --- | --- |
| AQ | MPPUS | 0.35 | < .001 |
| AQ | UADI-2 | 0.42 | < .001 |
| AQ | Tobacco (ASSIST) | − 0.02 | 0.633 |
| AQ | Alcohol (ASSIST) | 0.03 | 0.497 |
| K10 | MPPUS | 0.51 | < .001 |
| K10 | UADI-2 | 0.52 | < .001 |
| K10 | Tobacco (ASSIST) | 0.20 | < .001 |
| K10 | Alcohol (ASSIST) | 0.23 | < .001 |
