# Supplementary material for: Association between Problematic Internet and Mobile Phone Use, autistic traits, and psychological distress among adults: A cross-sectional survey
Source: PLOS Ment Health. 2026 Jun 2;3(6):e0000524. doi: 10.1371/journal.pmen.0000524 (PMC13229353; doi:10.1371/journal.pmen.0000524)
Supplement: S11 Table — (DOCX) [file pmen.0000524.s011.docx]

**Association Between Problematic Internet and Mobile Phone Use, Autistic Traits, and Psychological Distress Among Adults: A Cross-Sectional Survey**

Matilda Floris, Claudio Gentili

# **S11 Table (A). ANCOVA: Alcohol use (ASSIST).**

| Predictors | Degree of freedom | Sum of squares | Mean square | F value | η² | 95% CI | *p value* |
| --- | --- | --- | --- | --- | --- | --- | --- |
| Autistic traits | 1 | 20.734 | 20.734 | 0.727 | .002 | [0.00, 1.00] | 0.394 |
| K10 | 1 | 666.799 | 666.799 | 23.388 | .06 | [0.03, 1.00] | < .001 |
| Age group | 3 | 288.443 | 96.148 | 3.372 | .03 | [0.00, 1.00] | 0.019 |
| Education level | 4 | 135.206 | 33.802 | 1.186 | .01 | [0.00, 1.00] | 0.317 |
| Origin | 5 | 111.978 | 22.396 | 0.786 | .01 | [0.00, 1.00] | 0.561 |
| Marital status | 5 | 247.374 | 49.475 | 1.735 | .02 | [0.00, 1.00] | 0.126 |
| Housing | 5 | 32.962 | 6.592 | 0.231 | .003 | [0.00, 1.00] | 0.949 |
| Occupation | 5 | 384.462 | 76.892 | 2.697 | .04 | [0.00, 1.00] | 0.021 |
| Economic level | 6 | 86.440 | 14.407 | 0.505 | .008 | [0.00, 1.00] | 0.804 |
| Phone | 3 | 112.310 | 37.437 | 1.313 | .01 | [0.00, 1.00] | 0.270 |
| Use | 5 | 190.602 | 38.120 | 1.337 | .02 | [0.00, 1.00] | 0.248 |
| Social | 4 | 71.883 | 17.971 | 0.630 | .007 | [0.00, 1.00] | 0.641 |
| Chronic disease | 1 | 95.567 | 95.567 | 3.352 | .009 | [0.00, 1.00] | 0.068 |
| Psychological diagnosis | 1 | 0.829 | 0.829 | 0.029 | .000 | [0.00, 1.00] | 0.865 |
| Issues with justice | 2 | 170.024 | 85.012 | 2.982 | .02 | [0.00, 1.00] | 0.052 |
| Familiarity | 2 | 49.153 | 24.576 | 0.862 | .005 | [0.00, 1.00] | 0.423 |
| Trauma | 2 | 11.020 | 5.510 | 0.193 | .001 | [0.00, 1.00] | 0.824 |
| Residuals | 362 | 10,320.836 | 28.511 |  |  |  |  |

**S11.A.1.** Post-hoc Tukey Test: Age group

| Contrast | Estimate | Standard Error | Degree of freedom | t ratio | *p value* |
| --- | --- | --- | --- | --- | --- |
| 18–24 - 25–36 | 1.477 | 1.123 | 362 | 1.316 | 0.553 |
| 18–24 - 37–49 | 0.534 | 1.418 | 362 | 0.377 | 0.982 |
| 18–24 - 50–65 | 1.476 | 1.487 | 362 | 0.992 | 0.754 |
| 25–36 - 37–49 | -0.943 | 0.916 | 362 | -1.029 | 0.732 |
| 25–36 - 50–65 | -0.002 | 1.057 | 362 | -0.001 | 1 |
| 18–24 - 25–36 | 0.941 | 0.868 | 362 | 1.085 | 0.699 |

## **S11.A.2.** Post-hoc Tukey Test: Occupation

| Contrast | Estimate | Standard Error | Degree of freedom | t ratio | *p value* |
| --- | --- | --- | --- | --- | --- |
| Retired - Student | 5.119 | 2.278 | 362 | 2.247 | 0.219 |
| Retired - Student and worker | 3.109 | 2.226 | 362 | 1.397 | 0.729 |
| Retired - Unable to work | 1.744 | 6.036 | 362 | 0.289 | 1 |
| Retired - Unemployed | 5.590 | 2.455 | 362 | 2.277 | 0.206 |
| Retired - Worker | 2.361 | 1.941 | 362 | 1.216 | 0.829 |
| Student - Student and worker | -2.010 | 1.173 | 362 | -1.714 | 0.523 |
| Student - Unable to work | -3.375 | 5.791 | 362 | -0.583 | 0.992 |
| Student - Unemployed | 0.471 | 1.840 | 362 | 0.256 | 1 |
| Student - Worker | -2.758 | 1.181 | 362 | -2.335 | 0.183 |
| Student and worker - Unable to work | -1.365 | 5.783 | 362 | -0.236 | 1 |
| Student and worker - Unemployed | 2.480 | 1.818 | 362 | 1.364 | 0.748 |
| Student and worker - Worker | -0.749 | 1.101 | 362 | -0.680 | 0.984 |
| Unable to work - Unemployed | 3.846 | 5.869 | 362 | 0.655 | 0.987 |
| Unable to work - Worker | 0.617 | 5.676 | 362 | 0.109 | 1 |
| Unemployed - Worker | -3.229 | 1.522 | 362 | -2.121 | 0.279 |

# **S11 Table (B).** **ANCOVA: Tabacco use (ASSIST)**

| Predictor | Degree of freedom | Sum of squares | Mean square | F value | η² | 95% CI | *p value* |
| --- | --- | --- | --- | --- | --- | --- | --- |
| Autistic traits | 1 | 113.741 | 113.741 | 1.545 | .004 | [0.00, 1.00] | 0.215 |
| K10 | 1 | 2,899.102 | 2,899.102 | 39.377 | .10 | [0.05, 1.00] | < .001 |
| Age group | 3 | 1,898.514 | 632.838 | 8.595 | .07 | [0.03, 1.00] | < .001 |
| Education level | 4 | 235.560 | 58.890 | 0.800 | .009 | [0.00, 1.00] | 0.526 |
| Origin | 5 | 588.831 | 117.766 | 1.600 | .02 | [0.00, 1.00] | 0.159 |
| Marital status | 5 | 877.797 | 175.559 | 2.385 | .03 | [0.00, 1.00] | 0.038 |
| Housing | 5 | 333.115 | 66.623 | 0.905 | .01 | [0.00, 1.00] | 0.478 |
| Occupation | 5 | 742.234 | 148.447 | 2.016 | .03 | [0.00, 1.00] | 0.076 |
| Economic level | 6 | 314.196 | 52.366 | 0.711 | .01 | [0.00, 1.00] | 0.641 |
| Phone | 3 | 106.422 | 35.474 | 0.482 | .004 | [0.00, 1.00] | 0.695 |
| Use | 5 | 836.392 | 167.278 | 2.272 | .03 | [0.00, 1.00] | 0.047 |
| Social | 4 | 377.481 | 94.370 | 1.282 | .01 | [0.00, 1.00] | 0.277 |
| Chronic disease | 1 | 21.876 | 21.876 | 0.297 | .001 | [0.00, 1.00] | 0.586 |
| Psychological diagnosis | 1 | 1.445 | 1.445 | 0.020 | .000 | [0.00, 1.00] | 0.889 |
| Issues with justice | 2 | 51.535 | 25.768 | 0.350 | .002 | [0.00, 1.00] | 0.705 |
| Familiarity | 2 | 464.844 | 232.422 | 3.157 | .02 | [0.00, 1.00] | 0.044 |
| Trauma | 2 | 109.581 | 54.791 | 0.744 | .004 | [0.00, 1.00] | 0.476 |
| Residuals | 362 | 26,652.216 | 73.625 |  |  |  |  |

## **S11.B.1.** Post-hoc Tukey Test: Age group

| Contrast | Estimate | Standard Error | Degree of freedom | t ratio | *p value* |
| --- | --- | --- | --- | --- | --- |
| 18–24 - 25–36 | 1.722 | 1.804 | 362 | 0.955 | 0.775 |
| 18–24 - 37–49 | 4.970 | 2.278 | 362 | 2.181 | 0.130 |
| 18–24 - 50–65 | 4.899 | 2.390 | 362 | 2.050 | 0.172 |
| 25–36 - 37–49 | 3.247 | 1.472 | 362 | 2.206 | 0.123 |
| 25–36 - 50–65 | 3.177 | 1.699 | 362 | 1.870 | 0.243 |
| 37–49 - 50–65 | -0.070 | 1.394 | 362 | -0.050 | 1 |

## **S11.B.2.** Post-hoc Tukey Test: Marital status

| Contrast | Estimate | Standard Error | Degree of freedom | t ratio | *p value* |
| --- | --- | --- | --- | --- | --- |
| Divorced - Married | 0.453 | 3.209 | 362 | 0.141 | 1 |
| Divorced - Relationship | -2.516 | 3.246 | 362 | -0.775 | 0.972 |
| Divorced - Separated | -4.057 | 4.049 | 362 | -1.002 | 0.917 |
| Divorced - Single | -1.258 | 3.349 | 362 | -0.376 | 0.999 |
| Divorced - Widowed | 3.431 | 6.942 | 362 | 0.494 | 0.996 |
| Married - Relationship | -2.969 | 1.430 | 362 | -2.076 | 0.302 |
| Married - Separated | -4.511 | 3.108 | 362 | -1.451 | 0.695 |
| Married - Single | -1.711 | 1.867 | 362 | -0.917 | 0.942 |
| Married - Widowed | 2.978 | 6.432 | 362 | 0.463 | 0.997 |
| Relationship - Separated | -1.541 | 3.051 | 362 | -0.505 | 0.996 |
| Relationship - Single | 1.258 | 1.380 | 362 | 0.912 | 0.943 |
| Relationship - Widowed | 5.947 | 6.489 | 362 | 0.916 | 0.942 |
| Separated - Single | 2.799 | 3.077 | 362 | 0.910 | 0.944 |
| Separated - Widowed | 7.488 | 6.933 | 362 | 1.080 | 0.889 |
| Single - Widowed | 4.689 | 6.544 | 362 | 0.716 | 0.980 |

## **S11.B.3.** Post-hoc Tukey Test: Use

| Contrast | Estimate | Standard Error | Degree of freedom | t ratio | *p value* |
| --- | --- | --- | --- | --- | --- |
| Communication - Games | -3.453 | 2.813 | 362 | -1.227 | 0.823 |
| Communication - Internet navigation | 1.613 | 5.273 | 362 | 0.306 | 1 |
| Communication - Other | 2.108 | 1.571 | 362 | 1.342 | 0.761 |
| Communication - Shopping | -3.302 | 2.452 | 362 | -1.347 | 0.759 |
| Communication - (Social-network) | 1.631 | 1.099 | 362 | 1.484 | 0.675 |
| Games - Internet navigation | 5.066 | 5.941 | 362 | 0.853 | 0.957 |
| Games - Other | 5.561 | 3.112 | 362 | 1.787 | 0.475 |
| Games - Shopping | 0.150 | 3.621 | 362 | 0.042 | 1 |
| Games - (Social-network) | 5.083 | 2.761 | 362 | 1.841 | 0.441 |
| Internet navigation - Other | 0.495 | 5.425 | 362 | 0.091 | 1 |
| Internet navigation - Shopping | -4.915 | 5.722 | 362 | -0.859 | 0.956 |
| Internet navigation - (Social-network) | 0.018 | 5.315 | 362 | 0.003 | 1 |
| Other - Shopping | -5.410 | 2.774 | 362 | -1.950 | 0.373 |
| Other - (Social-network) | -0.477 | 1.681 | 362 | -0.284 | 1 |
| Shopping - (Social-network) | 4.933 | 2.530 | 362 | 1.949 | 0.374 |

## **S11.B.4.** Post-hoc Tukey Test: Familiarity

| Contrast | Estimate | Standard Error | Degree of freedom | t ratio | *p value* |
| --- | --- | --- | --- | --- | --- |
| No - Yes, behavioural addiction | 1.397 | 3.143 | 362 | 0.445 | 0.897 |
| No - Yes, substance addiction | -2.986 | 1.293 | 362 | -2.309 | 0.056 |
| Yes, behavioural addiction - Yes, substance addiction | -4.383 | 3.341 | 362 | -1.312 | 0.390 |

# **S11 Table (C).** **ANCOVA: Internet use (UADI-2)**

| Predictor | Degree of freedom | Sum of squares | Mean square | F value | η² | 95% CI | *p value* |
| --- | --- | --- | --- | --- | --- | --- | --- |
| Autistic traits | 1 | 8,326.819 | 8,326.819 | 61.667 | .15 | [0.09, 1.00] | < .001 |
| K10 | 1 | 20,559.619 | 20,559.619 | 152.260 | .30 | [0.23, 1.00] | < .001 |
| Age group | 3 | 8,905.881 | 2,968.627 | 21.985 | .15 | [0.10, 1.00] | < .001 |
| Education level | 4 | 261.973 | 65.493 | 0.485 | .005 | [0.00, 1.00] | 0.747 |
| Origin | 5 | 671.735 | 134.347 | 0.995 | .01 | [0.00, 1.00] | 0.421 |
| Marital status | 5 | 1,449.446 | 289.889 | 2.147 | .03 | [0.00, 1.00] | 0.059 |
| Housing | 5 | 1,046.057 | 209.211 | 1.549 | .02 | [0.00, 1.00] | 0.174 |
| Occupation | 5 | 742.582 | 148.516 | 1.100 | .01 | [0.00, 1.00] | 0.360 |
| Economic level | 6 | 1,311.642 | 218.607 | 1.619 | .03 | [0.00, 1.00] | 0.141 |
| Phone | 3 | 4,365.819 | 1,455.273 | 10.777 | .08 | [0.04, 1.00] | < .001 |
| Use | 5 | 1,290.440 | 258.088 | 1.911 | .03 | [0.00, 1.00] | 0.092 |
| Social | 4 | 2,520.447 | 630.112 | 4.666 | .05 | [0.01, 1.00] | 0.001 |
| Chronic disease | 1 | 417.600 | 417.600 | 3.093 | .008 | [0.00, 1.00] | 0.079 |
| Psychological diagnosis | 1 | 3.922 | 3.922 | 0.029 | .008 | [0.00, 1.00] | 0.865 |
| Issues with justice | 2 | 829.257 | 414.629 | 3.071 | .02 | [0.00, 1.00] | 0.048 |
| Familiarity | 2 | 88.954 | 44.477 | 0.329 | .002 | [0.00, 1.00] | 0.720 |
| Trauma | 2 | 216.855 | 108.427 | 0.803 | .004 | [0.00, 1.00] | 0.449 |
| Residuals | 362 | 48,880.685 | 135.030 |  |  |  |  |

## **S11.C.1.** Post-hoc Tukey Test: Age group

| Contrast | Estimate | Standard Error | Degree of freedom | t ratio | *p value* |
| --- | --- | --- | --- | --- | --- |
| 18–24 - 25–36 | 1.121 | 2.443 | 362 | 0.459 | 0.968 |
| 18–24 - 37–49 | 2.970 | 3.085 | 362 | 0.962 | 0.771 |
| 18–24 - 50–65 | 2.406 | 3.237 | 362 | 0.743 | 0.880 |
| 25–36 - 37–49 | 1.849 | 1.993 | 362 | 0.928 | 0.790 |
| 25–36 - 50–65 | 1.285 | 2.301 | 362 | 0.558 | 0.944 |
| 37–49 - 50–65 | -0.564 | 1.888 | 362 | -0.299 | 0.991 |

## **S11.C.2.** Post-hoc Tukey Test: Phone

| Contrast | Estimate | Standard Error | Degree of freedom | t ratio | *p value* |
| --- | --- | --- | --- | --- | --- |
| 2-5 hours - 5-8 hours | -1.165 | 2.059 | 362 | -0.566 | 0.942 |
| 2-5 hours - 8+ hours | -1.297 | 4.445 | 362 | -0.292 | 0.991 |
| 2-5 hours - Less than 2 hours | 5.569 | 1.569 | 362 | 3.550 | 0.002 |
| 5-8 hours - 8+ hours | -0.131 | 4.499 | 362 | -0.029 | 1 |
| 5-8 hours - Less than 2 hours | 6.734 | 2.460 | 362 | 2.737 | 0.033 |
| 8+ hours - Less than 2 hours | 6.866 | 4.637 | 362 | 1.481 | 0.450 |

## **S11.C.3.** Post-hoc Tukey Test: Social

| Contrast | Estimate | Standard Error | Degree of freedom | t ratio | *p value* |
| --- | --- | --- | --- | --- | --- |
| 2 hours - 5 hours | -11.404 | 4.764 | 362 | -2.394 | 0.119 |
| 2 hours - 7 hours | 6.451 | 2.346 | 362 | 2.750 | 0.049 |
| 2 hours - 7+ hours | -3.042 | 1.701 | 362 | -1.788 | 0.382 |
| 2 hours - Never | 3.550 | 6.127 | 362 | 0.579 | 0.978 |
| 5 hours - 7 hours | 17.855 | 5.348 | 362 | 3.338 | 0.008 |
| 5 hours - 7+ hours | 8.362 | 4.560 | 362 | 1.834 | 0.356 |
| 5 hours - Never | 14.954 | 6.740 | 362 | 2.219 | 0.175 |
| 7 hours - 7+ hours | -9.494 | 2.867 | 362 | -3.311 | 0.009 |
| 7 hours - Never | -2.902 | 6.536 | 362 | -0.444 | 0.992 |
| 7+ hours - Never | 6.592 | 5.987 | 362 | 1.101 | 0.806 |

## **S11.C.4.** Post-hoc Tukey Test: Issues with justice

| Contrast | Estimate | Standard Error | Degree of freedom | t ratio | *p value* |
| --- | --- | --- | --- | --- | --- |
| No - Prisoned | -15.050 | 7.435 | 362 | -2.024 | 0.108 |
| No - Yes civil | 6.736 | 5.087 | 362 | 1.324 | 0.383 |
| Prisoned - Yes civil | 21.786 | 8.980 | 362 | 2.426 | 0.042 |

# **S11 Table (D)**. **ANCOVA: Mobile phone use (MPPUS)**

| Predictor | Degree of freedom | Sum of squares | Mean square | F value | η² | 95% CI | *p value* |
| --- | --- | --- | --- | --- | --- | --- | --- |
| Autistic traits | 1 | 4,877.439 | 4,877.439 | 33.251 | .08 | [0.04, 1.00] | < .001 |
| K10 | 1 | 23,062.116 | 23,062.116 | 157.221 | .30 | [0.24, 1.00] | < .001 |
| Age group | 3 | 6,903.071 | 2,301.024 | 15.687 | .12 | [0.06, 1.00] | < .001 |
| Education level | 4 | 548.630 | 137.158 | 0.935 | .01 | [0.00, 1.00] | 0.444 |
| Origin | 5 | 341.940 | 68.388 | 0.466 | .006 | [0.00, 1.00] | 0.801 |
| Marital status | 5 | 1,048.506 | 209.701 | 1.430 | .02 | [0.00, 1.00] | 0.213 |
| Housing | 5 | 420.808 | 84.162 | 0.574 | .008 | [0.00, 1.00] | 0.720 |
| Occupation | 5 | 287.296 | 57.459 | 0.392 | .005 | [0.00, 1.00] | 0.854 |
| Economic level | 6 | 724.900 | 120.817 | 0.824 | .01 | [0.00, 1.00] | 0.552 |
| Phone | 3 | 8,912.887 | 2,970.962 | 20.254 | .014 | [0.09, 1.00] | < .001 |
| Use | 5 | 1,611.842 | 322.368 | 2.198 | .03 | [0.00, 1.00] | 0.054 |
| Social | 4 | 2,552.865 | 638.216 | 4.351 | .05 | [0.01, 1.00] | 0.002 |
| Chronic disease | 1 | 292.724 | 292.724 | 1.996 | .005 | [0.00, 1.00] | 0.159 |
| Psychological diagnosis | 1 | 4.883 | 4.883 | 0.033 | .000 | [0.00, 1.00] | 0.855 |
| Issues with justice | 2 | 1,183.141 | 591.571 | 4.033 | .02 | [0.00, 1.00] | 0.019 |
| Familiarity | 2 | 324.085 | 162.042 | 1.105 | .006 | [0.00, 1.00] | 0.332 |
| Trauma | 2 | 88.483 | 44.241 | 0.302 | .001 | [0.00, 1.00] | 0.740 |
| Residuals | 362 | 53,100.415 | 146.686 |  |  |  |  |

## **S11.D.1.** Post-hoc Tukey Test: Age group

| Contrast | Estimate | Standard Error | Degree of freedom | t ratio | *p value* |
| --- | --- | --- | --- | --- | --- |
| 18–24 - 25–36 | -0.800 | 2.546 | 362 | -0.314 | 0.989 |
| 18–24 - 37–49 | 0.496 | 3.216 | 362 | 0.154 | 0.999 |
| 18–24 - 50–65 | 0.136 | 3.374 | 362 | 0.040 | 1 |
| 25–36 - 37–49 | 1.296 | 2.077 | 362 | 0.624 | 0.924 |
| 25–36 - 50–65 | 0.936 | 2.398 | 362 | 0.390 | 0.980 |
| 37–49 - 50–65 | -0.360 | 1.968 | 362 | -0.183 | 0.998 |

## **S11.D.2.** Post-hoc Tukey Test: Phone

| Contrast | Estimate | Standard Error | Degree of freedom | t ratio | *p value* |
| --- | --- | --- | --- | --- | --- |
| 2-5 hours - 5-8 hours | -3.950 | 2.146 | 362 | -1.840 | 0.256 |
| 2-5 hours - 8+ hours | -2.957 | 4.633 | 362 | -0.638 | 0.920 |
| 2-5 hours - Less than 2 hours | 7.230 | 1.635 | 362 | 4.422 | < .001 |
| 5-8 hours - 8+ hours | 0.993 | 4.689 | 362 | 0.212 | 0.997 |
| 5-8 hours - Less than 2 hours | 11.180 | 2.564 | 362 | 4.360 | < .001 |
| 8+ hours - Less than 2 hours | 10.187 | 4.833 | 362 | 2.108 | 0.153 |

## **S11.D.3.** Post-hoc Tukey Test: Social

| Contrast | Estimate | Standard Error | Degree of freedom | t ratio | *p value* |
| --- | --- | --- | --- | --- | --- |
| 2 hours - 5 hours | -17.373 | 4.965 | 362 | -3.499 | 0.005 |
| 2 hours - 7 hours | 4.446 | 2.445 | 362 | 1.818 | 0.365 |
| 2 hours - 7+ hours | -4.468 | 1.773 | 362 | -2.520 | 0.089 |
| 2 hours - Never | -5.458 | 6.386 | 362 | -0.855 | 0.913 |
| 5 hours - 7 hours | 21.819 | 5.575 | 362 | 3.914 | 0.001 |
| 5 hours - 7+ hours | 12.905 | 4.753 | 362 | 2.715 | 0.054 |
| 5 hours - Never | 11.915 | 7.024 | 362 | 1.696 | 0.438 |
| 7 hours - 7+ hours | -8.915 | 2.988 | 362 | -2.983 | 0.025 |
| 7 hours - Never | -9.904 | 6.813 | 362 | -1.454 | 0.593 |
| 7+ hours - Never | -0.989 | 6.240 | 362 | -0.159 | 1 |

## **S11.D.4.** Post-hoc Tukey Test: Issues with justice

| Contrast | Estimate | Standard Error | Degree of freedom | t ratio | *p value* |
| --- | --- | --- | --- | --- | --- |
| No - Prisoned | -20.410 | 7.749 | 362 | -2.634 | 0.024 |
| No - Yes civil | 2.453 | 5.302 | 362 | 0.463 | 0.889 |
| Prisoned - Yes civil | 22.862 | 9.360 | 362 | 2.443 | 0.040 |
