# Supplementary material for: Association between Problematic Internet and Mobile Phone Use, autistic traits, and psychological distress among adults: A cross-sectional survey
Source: PLOS Ment Health. 2026 Jun 2;3(6):e0000524. doi: 10.1371/journal.pmen.0000524 (PMC13229353; doi:10.1371/journal.pmen.0000524)
Supplement: S12 Table — (DOCX) [file pmen.0000524.s012.docx]

**Association Between Problematic Internet and Mobile Phone Use, Autistic Traits, and Psychological Distress Among Adults: A Cross-Sectional Survey**

Matilda Floris, Claudio Gentili

**S12 Table. Hurdle Models for ASSIST-Tobacco and ASSIST-Alcohol scores.**

|  | **ASSIST - Tobacco** | | | **ASSIST - Alcohol** | | |
| --- | --- | --- | --- | --- | --- | --- |
| Predictors | 𝛽 (Estimate) | CI | *p* | 𝛽 (Estimate) | CI | *p* |
| (Intercept) | 1.91 | 1.49 – 2.33 | **<0.001** | 1.41 | 1.07 – 1.74 | **<0.001** |
| AQ trait [Low autistic traits] | 0.31 | 0.02 – 0.60 | **0.033** | 0.20 | -0.02 – 0.43 | 0.079 |
| K10 | 0.02 | 0.01 – 0.03 | **0.001** | 0.02 | 0.01 – 0.03 | **0.001** |
| **Zero Hurdle Model** | | | | | | |
| (Intercept) | -1.56 | -2.42 – -0.70 | **<0.001** | -0.17 | -1.24 – 0.90 | 0.750 |
| AQ trait [Low autistic traits] | 0.41 | -0.16 – 0.99 | 0.159 | 0.20 | -0.51 – 0.91 | 0.582 |
| K10 | 0.05 | 0.03 – 0.08 | **<0.001** | 0.06 | 0.03 – 0.10 | **0.001** |
| Observations | 420 | | | 420 | | |
| R^2^ / R^2^ adjusted | 0.894 / 0.893 | | | 0.608 / 0.605 | | |

Note. The table reports raw unstandardized coefficients (β) and 95% confidence intervals (CI). The Hurdle model is composed of two parts: (1) the Count Model (truncated negative binomial with log link), which estimates the association with the intensity of substance use among users (scores > 0); and (2) the Zero Hurdle Model (binomial with logit link), which estimates the probability of being a user versus a non-user. Psychological distress (K10) was included as a covariate. Autistic traits was included as main independent variables, with “high autistic traits” as the reference category.
