# Supplementary material for: Association between Problematic Internet and Mobile Phone Use, autistic traits, and psychological distress among adults: A cross-sectional survey
Source: PLOS Ment Health. 2026 Jun 2;3(6):e0000524. doi: 10.1371/journal.pmen.0000524 (PMC13229353; doi:10.1371/journal.pmen.0000524)
Supplement: Attachment — Submitted filename: Response to Reviewers_0402.docx [file pmen.0000524.s014.docx]

**Point-by-point Authors Response to Journal Requirements and Reviewer Comments**

**Journal Requirement**

1. Please amend your detailed Financial Disclosure statement. This is published with the article. It must therefore be completed in full sentences and contain the exact wording you wish to be published.

*The Financial Disclosure statement has been added to the manuscript: “M.F. was funded by a grant from the National Recovery and Resilience Plan (PNRR) of the European Union (NextGeneration EU). The funders had no role in the study design, data collection and analysis, decision to publish, or preparation of the manuscript.”*

2. Please provide separate figure files in .tif or .eps format.

*We used the PLOS’s NAAS tool to convert Figure 1 to the TIFF format, and we uploaded the converted file during resubmission.*

3. We have noticed that you have uploaded Supporting Information files, but you have not included a list of legends. Please add a full list of legends for your Supporting Information files after the references list.

*We have included a list of legends for all Supporting Information files after the references list.*

4. In the online submission form, you indicated that “The collected data included sensitive informations (e.g., IP addresses, response IDs, medical and psychological diagnosis, traumatic events, justice-related experiences). Therefore, the dataset is available from the corresponding author upon motivated request.”.

*We have made the anonymized dataset, the analysis code, and the codebook available on the OSF website (*[*https://osf.io/yvrfq/overview*](https://osf.io/yvrfq/overview)*).*

**Reviewer comments:**

**Reviewer #1:** The manuscript describes the analysis of a cross-sectional survey on Italian adults (n=420), examining associations between autistic traits, problematic internet/mobile phone use (PIU/PMPU), psychological distress, and substance use. The results show that autistic traits correlated with PIU/PMPU, and psychological distress correlates with all outcomes. The manuscript is clearly written, and the topic is relevant for PLOS Mental Health. However, there are some methodological and reporting issues that should be addressed. I detail them below.

**Preregistration and data availability:**
The link to the preregistration does not work for me, so it is impossible to check: <https://osf.io/d2nbg>
We know from substantial literature now that datasets "available from the corresponding author upon motivated request" are often difficult, if not impossible, to access.
The authors are required to make available online the preregistration, as well as an **anonymised version of the dataset and the codes** used to analyse it.

*Thank you for your comments. We have addressed the issue related to the pre-registration link (*[*https://osf.io/d2nbg/overview?view_only=17adeb498f104e24976c4bf34948256a*](https://osf.io/d2nbg/overview?view_only=17adeb498f104e24976c4bf34948256a)*). Additionally, we have made the anonymized dataset and the analysis code available on the OSF website (*[*https://osf.io/yvrfq/overview*](https://osf.io/yvrfq/overview)*).*

**Generalisation and causal language in the discussion:**
The authors recognise some of the limitations e.g., that the sample is heavily skewed (80% female, 63% from Italian islands) and that "the cross-sectional design [...] limits generalizability and the causal interpretation of the results". However, the discussion moves toward causal language (e.g., “autistic traits may increase the vulnerability to develop PIU and PMPU”) and broadly generalises.

*We have revised several sentences in the Discussion section, particularly from page 30 (line 1), to limit causal language and overgeneralization, underlying limitations of the study.*

Related, convenience and especially snowball recruitment via social media may also be problematic. Given the topic (digital use), recruitment channels may have selected individuals already more online, more distressed, or more neurodivergent. This also requires more explicit acknowledgement.

*Thank you for raising this limitation of the study. We would like to clarify that only a minority of participants were recruited through social media, as the majority were recruited through word of mouth. However, as we acknowledge the limitations associated with this type of recruitment, we have added snowball sampling as limitation in the Limitation and future studies section. Nonetheless, we additional specify that the overall sample did not exhibit particularly elevated levels of PIU or PMPU; therefore, the type of recruitment is likely to have biased the results only partially.*

**Variables validity/Analysis:**
The authors do a good job in explaining and justifying their variable choices. Here are a few concerns:

The AQ sub-domains have "poor to acceptable internal consistency". E.g., the imagination subscale has α = .37. Would this instability propagate into the main predictor? Maybe an analysis with only the dimensions that show acceptable internal consistency could be presented to reassure about the robustness of the results in this respect?

*We understand your concern regarding the poor internal consistency (α = .37) of the AQ Imagination subscale and, therefore, we re-rerun the main analysis excluding this subscale.*

*Specifically, items of Imagination subscale were removed and the AQ total score was recalculated. The new AQ total score showed a global Cronbach’s α of .82, as the original analysis, indicating a good internal consistency. We re-run Spearman’s correlations between corrected AQ score (without Imagination subscale) and each dependent variable (UADI-2, MPPUS, ASSIST-alcohol and ASSIST-tobacco) across age groups. The table below summarized both the new correlations based on AQ corrected score and the original correlations reported in Table 3 of the manuscript. Although minor numerical differences emerged, the overall pattern of results is unchanged.*

| **Age groups** | **Variables** | **Rho (original)** | **p-value (original)** | **Rho (without Imagination subscale)** | **p-value (without Imagination subscale)** | **n** |
| --- | --- | --- | --- | --- | --- | --- |
| **18–24** | ASSIST - Alcohol | -0.17 | 0.073 | -0.14 | 0.148 | 114 |
| **18–24** | ASSIST – Tobacco | -0.19 | 0.049 | -0.19 | 0.046 | 114 |
| **18–24** | MPPUS | 0.41 | < .001 | 0.39 | < .001 | 114 |
| **18–24** | UADI2 | 0.46 | < .001 | 0.48 | < .001 | 114 |
| **25–36** | ASSIST - Alcohol | -0.04 | 0.691 | -0.05 | 0.583 | 107 |
| **25–36** | ASSIST – Tobacco | -0.06 | 0.555 | -0.03 | 0.772 | 107 |
| **25–36** | MPPUS | 0.42 | < .001 | 0.46 | < .001 | 107 |
| **25–36** | UADI2 | 0.50 | < .001 | 0.53 | < .001 | 107 |
| **37–49** | ASSIST - Alcohol | 0.06 | 0.515 | 0.07 | 0.446 | 106 |
| **37–49** | ASSIST – Tobacco | -0.09 | 0.369 | -0.05 | 0.576 | 106 |
| **37–49** | MPPUS | 0.24 | 0.015 | 0.26 | 0.007 | 106 |
| **37–49** | UADI2 | 0.30 | 0.002 | 0.33 | < .001 | 106 |
| **50–65** | ASSIST - Alcohol | 0.14 | 0.194 | 0.13 | 0.213 | 93 |
| **50–65** | ASSIST – Tobacco | 0.07 | 0.517 | 0.07 | 0.487 | 93 |
| **50–65** | MPPUS | 0.12 | 0.238 | 0.19 | 0.071 | 93 |
| **50–65** | UADI2 | 0.22 | 0.033 | 0.29 | 0.006 | 93 |

*MANCOVA were not performed using the AQ corrected score, as this analysis required categorical grouping (AQ traits). We cannot compute AQ traits without the Imagination subscale, as the established cut-off (≥ 26) does not retain the same validity. Thus, multivariate multiple regression (MMR) was re-run treating the correct AQ score as a continuous variable. The results align with those of the original analysis:*

- *PIU (UADI-2) was significantly predicted by both AQ (β = 0.69, SE = 0.08, t(417) = 8.41, p < .001) and K10 (β = 0.72, SE = 0.11, t(417) = 6.16, p < .001), as in the original analysis*
- *PMPU (MPPUS) was significantly predicted by both AQ (β = 0.42, SE = 0.11, t(417) = 3.62, p < .001) and K10 (β = 0.83, SE = 0.09, t(417) = 9.18, p < .001), as in the original analysis*
- *Alcohol use (ASSIST) was significantly predicted by only K10 (β = 0.16, SE = 0.03, t(417) = 4.38, p < .001), as in the original analysis*
- *Tobacco use (ASSIST) was significantly predicted by only K10 (β = 0.36, SE = 0.06, t(417) = 6.11, p < .001), as in the original analysis*

*These results support the robustness of the main findings, indicating that the poor internal consistency of the AQ Imaginal subscale did not substantially affect the results. Since the results were virtually unchanged, and in order to ensure better comparability with the available literature and to maintain the MANOVA analysis, we decided to retain the original analysis in the manuscript and not to reported this latter. Should the reviewer suggest including it, we will add it as supplementary sensitivity analysis.*

Given the absence of data, it is impossible to check, but it is plausible that ASSIST alcohol and ASSIST tobacco are zero-inflated variables. Even if the authors use non-parametric tests, they assume standard continuous values, which can create a problem.

*We thank the reviewer for raising this important point regarding the potential zero‐inflation of ASSIST alcohol and ASSIST tobacco scores. We agree that ASSIST alcohol, and particularly ASSIST tobacco, show a high proportion of zero scores. Specifically, 21% of participants scored 0 on ASSIST‐alcohol (n= 90), whereas 51% scored 0 on ASSIST‐tobacco (n= 216). These results were currently included in Supporting Information S7.*

*As you said, associations between ASSIST scores and other variables were examined using non‐parametric tests (i.e., Kruskal–Wallis tests and Spearman’s correlations), which do not assume normality and are more robust to skewed and zero‐inflated distributions. Nonetheless, we acknowledge that the MANCOVA treated ASSIST scores as continuous outcomes, which may be suboptimal given the distributional characteristics of ASSIST-tobacco in particular. Therefore, we performed follow-up Hurdle Models analyses (Mullahy, 1986; Cragg, 1971), tailored for zero-inflated data, to separately estimate the probability of tobacco and alcohol use (0 vs >0) and the intensity of use among users.*

*The zero Hurdle Model results indicated that autistic traits did not impact the probability of becoming a user of tobacco (𝛽= 0.41, p = .16) or alcohol (𝛽= 0.20, p = .58). On the contrary, psychological distress remained a highly significant predictor both of tobacco (𝛽= 0.05, p < .001) and alcohol use (𝛽= 0.06, p < .001).*

*The results of the count model showed that, within tobacco users, low autistic traits significantly predicted the intensity of tobacco use (𝛽= 0.31, p = .03). In other words, within tobacco users, those with lower autistic traits showed higher tobacco use, compared to those with high autistic traits. This result is connected to what emerged from correlational and MMR analyses (i.e., that autistic traits and tobacco use were significantly associated) and, therefore, we specify it in the Discussion section (page 29). On the contrary, within alcohol users, low autistic traits were not a significant predictor of alcohol use (𝛽= 0.20, p = .07). In the count model, psychological distress (K10) was significantly associated with higher substance use (both 𝛽= 0.02, p < .001) within both tobacco and alcohol users.*

*Compared to the original MANCOVA results (i.e., both autistic traits and psychological distress significantly predicted outcomes), the results of the Hurdle models were consistent. As proposed in the Discussion section, autistic traits may be related more to PIU/PMPU while psychological distress can be broadly linked additionally to substance use. The Hurdle models further supported our findings, indicating that psychological distress increases the probability of becoming a substance user and the intensity of use. Thus, we retained the original MANCOVA model as the main analysis and included the Hurdle models as a sensitivity analysis in the manuscript, reporting the corresponding table in the Supporting Materials (S13), to ensure the robustness of our conclusions.*

Psychological distress, PIU, and PMPU are all moderately to strongly interrelated. Using them simultaneously in MANCOVA without addressing multicollinearity risks unstable estimates.

*We acknowledge the overlap between PIU and PMPU, and we have already included it as a limitation of the study. However, we have checked multicollinearity among outcomes through (a) Spearman correlation between them and (b) variance inflation factors (VIFs). Spearman correlations indicated weak associations between substance use measures and Internet-related behaviors:*

- *ASSIST-Alcohol – ASSIST-Tobacco: r = .34;*
- *ASSIST- Alcohol – UADI-2: r = .26;*
- *ASSIST- Alcohol – MPPUS: r = .21;*
- *ASSIST - Tabacco – UADI-2: r = .17;*
- *ASSIST-Tabacco – MPPUS: r = .13.*

*As expected, UADI-2 and MPPUS were more strongly correlated (r = .79), confirming the overlap between PIU and PMPU. However, the two instruments differ in their conceptual focus: MPPUS specifically assess mobile phone use and related distress, while UADI-2 evaluates PIU more broadly, with no item directly referring to mobile phone use.*

*VIF results were below the threshold of 5 (range = 1.03 – 2.65, see table below) (James et al., 2013), indicating that multicollinearity was not sufficiently severe to compromise the stability of MANCOVA. On this basis, and given the robustness of Pillai’s trace, we have considered appropriate to include all four outcomes in the original MANCOVA model.*

*We have also added the multicollinearity results to the manuscript (page 23).*

**Table.** Variance inflation factors (VIFs)

| **Dependent variable** | **Predictor** | **VIF** |
| --- | --- | --- |
| ASSIST - Alcohol | | |
|  | UADI-2 | 2.64 |
|  | MPPUS | 2.63 |
|  | ASSIST-Tobacco | 1.03 |
| ASSIST - Tobacco | | |
|  | UADI-2 | 2.65 |
|  | MPPUS | 2.64 |
|  | ASSIST-Alcohol | 1.07 |
| MPPUS | | |
|  | ASSIST-Alcohol | 1.12 |
|  | ASSIST-Tobacco | 1.09 |
|  | UADI-2 | 1.08 |
| UADI-2 | | |
|  | ASSIST-Alcohol | 1.12 |
|  | ASSIST-Tobacco | 1.08 |
|  | MPPUS | 1.07 |

**Minor points:**

Double-check the references, e.g. "Elhai JD, Dvorak RD, Levine JC, Hall BJ. Problematic smartphone use..." is reported twice.

*Thank you, the references have now been corrected.*

Several tables and statistical results are reported in the manuscript, making it difficult to read. I would suggest selecting what the important ones are, and moving the rest to supplementary material or similar.

*Thank you for the suggestion. We have moved Table 2 (current S6) and part of the sociodemographic variables reported in Table 1 (current S4) to the Supporting Information. Additionally, we have removed the details of Tukey’s post hoc tests from the manuscript, that were now reported exclusively in the Supporting Information from S9 to S12.*

**Reviewer #2:** In this manuscript, the authors present an online cross-sectional survey (N=420) examining associations between autistic traits (AQ), problematic Internet use (UADI-2), problematic mobile phone use (MPPUS), psychological distress (K10), substance use (ASSIST), and age in an Italian community sample. Higher autistic traits and—more consistently—higher psychological distress are associated with greater PIU/PMPU, with younger adults showing the highest levels.
The topic is timely and the manuscript is generally clear and well structured. However, several points should be clarified to improve reproducibility and interpretation.

**Introduction**
• The authors should explicitly state how PIU and PMPU were defined and measured.

*In the Introduction (page 3, from line 24), PIU is defined as “preoccupation with and lack of control over diverse Internet-related behaviors* *– including general excessive use, gaming, smartphone use, and social networking – that lead to clinically significant distress or impairment in multiple areas of functioning.”. We now explicitly state that is the definition considered in the present study. The definition of PMPU is added on page 4 (line 11-12), as “difficulties in controlling time spent on mobile phones, which can negatively impact daily functioning”. Details on how PIU and PMPU were measured are provided in the Method section (from page 10, Survey paragraph). We prefer to keep the specific instruments in the Method section. The abstract has been updated to explicitly indicate how PIU and PMPU were assessed.*

• The authors should briefly introduce the I-PACE model (if used to guide interpretation) already in the Introduction, not only in the Discussion.

*We have moved the description of I-PACE model to the Introduction (page 7, Study paragraph).*

• The authors should clarify that the focus is on autistic traits in the general population, not clinical ASD diagnosis.

*We have made several modifications to the Introduction to strengthen the focus on autistic traits. In the paragraph “PIU, PMPU, autistic traits and psychological distress”, we also removed a reference to a study including only individuals with ASD (i.e., Eltahir et al. 2025). In the “PIU, PMPU, autistic traits and substance abuse”, we clarified that “the risk of substance abuse within the autistic population remains less clear, and studies involving individuals with autistic traits are lacking.”*

**Methods**
• The authors should briefly discuss likely selection effects from convenience/social-media recruitment and implications for prevalence estimates.

*Thank you for the comment, we have added it as a limit in the manuscript (page 32, lines 3-7).*

• The authors should more clearly report the deviation from preregistration regarding age grouping, and consider a sensitivity analysis using age as a continuous predictor/covariate.

*We thank the reviewer for this helpful suggestion. We have now explicitly reported the deviation from the pre-registration in a dedicated paragraph in the Methods section. In addition, we conducted a sensitivity analysis in which age was included as a continuous covariate in the MANCOVA model. This sensitivity MANCOVA showed that age, autistic traits, and K10 scores all had significant effects on the prediction of dependent variables. Thus, the association between autistic traits and the dependent variables remained robust and statistically significant when controlling for age as continuous variables. We add this sensitivity MANCOVA to the manuscript.*

**• The authors should clarify outcome classifications:**
o For UADI-2, the authors should specify whether “PIU prevalence” refers to misuse only or misuse + addiction, as current wording appears inconsistent with the categorical reporting.

*Thank you for raising this important point. We agree that the use of the terms “addiction” and “misuse” in the Table 2 and in the Dependent variable paragraph (page 13, line 3) could be confounding, even though these terms are those originally ones used in the standardization paper. To avoid this ambiguity, we removed the categorical classification of UADI-2 from Table 2 and revised the abstract to report PIU in terms of mean and standard deviation only. Throughout the manuscript, PIU prevalence (now “frequency”, see next point) now refers to descriptive statistics (i.e., mean and standard deviations) rather than categorical classifications.*

o For MPPUS, the authors should justify tertile-based “high use” as a proxy for PMPU prevalence and emphasize continuous analyses as primary, given sample-dependence.

*We thank the reviewer for this important methodological comment. We agree that tertile-based classification does not provide a true estimate of PMPU prevalence, as it is sample-dependent. Accordingly, we have replaced the term “prevalence” with “frequency” throughout the manuscript.*

*We also clarify that the main analyses involved MPPUS score as continuous variable. The tertile-based categorization is therefore retained only to describe the characteristics of the sample. For this reason, we have removed the tertile-based categorization from Table 2. In addition, we revised the abstract reporting mean and standard deviation of the MPPUS, rather than percentage of high use. Consistent changes were also made in Discussion section.*

• The authors should clarify why only a subsample completed the IAT, and whether this subsample differs from the full sample.

*Internet addiction test (IAT) was added later compared to the original set of questionnaires, in order to enhance comparability with the international literature. While the UADI-2 has been validated only in an Italian sample, the IAT is widely used internationally and allows cross-study comparisons. Therefore, only a subsample of participants completed IAT. Given that the IAT assesses PIU as UADI-2, the Ethical Committee of University of Padova did not require an amendment to the original ethical request. As the IAT was not part of original hypotheses, the pre-registration was not updated, and it was not included in the main analyses.*

*Participants who complete IAT did not differ from those who not complete it, as shown in the table below.*

***Table.*** *Sociodemographic and Internet and mobile phone use variables of the participants who complete IAT (n= 230) compared with those who did not complete IAT (n= 214).*

|  | **Samples** | |  |  |
| --- | --- | --- | --- | --- |
| **Variables** | **IAT**  n = 230 | **No IAT**  n = 214 | **Statistical Test** | ***p-value*** |
| **Gender** |  |  | Fisher’s Exact Test | 0.513 |
| Female | 177 (82%) | 165 (77%) |  |  |
| Male | 36 (17%) | 45 (21%) |  |  |
| Non binary | 1 (0.5%) | 1 (0.5%) |  |  |
| Prefer to not answer | 1 (0.5%) | 3 (1.5%) |  |  |
| **Age** |  |  | W = 25005 | 0.770 |
| Mean (SD) | 39 (14) | 39 (14) |  |  |
| Median | 38 | 37 |  |  |
| Min - Max | 18 - 75 | 18 - 65 |  |  |
| **Education level** |  |  | χ^2^ (4, N = 444) = 5.38) | 0.250 |
| High school | 80 (37%) | 90 (42%) |  |  |
| Master's degree | 49 (23%) | 49 (23%) |  |  |
| Bachelor's degree | 47 (22%) | 42 (20%) |  |  |
| Post-lauream | 31 (14%) | 19 (9%) |  |  |
| Middle school | 8 (4%) | 14 (6%) |  |  |
| **Citizenship** |  |  | Fisher’s Exact Test | 0.498 |
| Italian | 213 (99%) | 214 (100%) |  |  |
| European | 2 (1%) | 0 (0%) |  |  |
| **Origin** |  |  | Fisher’s Exact Test | 0.148 |
| Islands | 131 (61%) | 139 (65%) |  |  |
| North Italy | 50 (23%) | 55 (26%) |  |  |
| Central Italy | 15 (7.0%) | 11 (5%) |  |  |
| South Italy | 13 (6.0%) | 9 (4%) |  |  |
| Europe | 5 (2.5%) | 0 (0%) |  |  |
| Extra-Europe | 1 (0.5%) | 0 (0%) |  |  |
| **Marital status** |  |  | Fisher’s Exact Test | 0.743 |
| Relationship | 83 (39%) | 90 (42%) |  |  |
| Married | 66 (31%) | 72 (34%) |  |  |
| Single | 52 (24%) | 43 (20%) |  |  |
| Divorced | 6 (2.5%) | 3 (1.5%) |  |  |
| Separated | 6 (2.5%) | 5 (2%) |  |  |
| Widowed | 2 (1%) | 1 (0.5%) |  |  |
| **Housing** |  |  | Fisher’s Exact Test | 0.407 |
| With partner | 98 (46%) | 109 (51%) |  |  |
| With parents | 43 (20%) | 33 (15%) |  |  |
| Alone | 32 (15%) | 35 (16%) |  |  |
| With roommates | 24 (11%) | 27 (13%) |  |  |
| Other | 17 (7.5%) | 10 (5%) |  |  |
| University residence | 1 (0.5%) | 0 (0%) |  |  |
| **Occupation** |  |  | Fisher’s Exact Test | 0.678 |
| Worker | 131 (61%) | 132 (62%) |  |  |
| Student | 43 (20%) | 38 (18%) |  |  |
| Student and worker | 21 (10%) | 27 (13%) |  |  |
| Retired | 12 (5.5%) | 7 (3%) |  |  |
| Unemployed | 8 (3.5%) | 8 (3.5%) |  |  |
| Unable to work | 0 (0%) | 1 (0.5%) |  |  |
| **Mobile Phone** |  |  | χ^2^ (3, N = 444) = 0.67) | 0.880 |
| 2-5 hours | 122 (57%) | 124 (58%) |  |  |
| 5-8 hours | 30 (14%) | 24 (11%) |  |  |
| 8+ hours | 7 (3%) | 7 (3%) |  |  |
| Less than 2 hours | 56 (26%) | 57 (27%) |  |  |
| **Social network** |  |  | Fisher’s Exact Test | 0.923 |
| 2 hours | 131 (61%) | 134 (63%) |  |  |
| 5 hours | 5 (2.3%) | 4 (2.2%) |  |  |
| 7 hours | 21 (9.7%) | 16 (7.6%) |  |  |
| 7+ hours | 55 (26%) | 54 (25%) |  |  |
| Never | 3 (1%) | 4 (2.2%) |  |  |
| **Mobile Phone Use** |  |  | Fisher’s Exact Test | 0.519 |
| Communication | 113 (53%) | 98 (46%) |  |  |
| Games | 6 (2.5%) | 7 (3%) |  |  |
| Internet navigation | 0 (0%) | 3 (1.5%) |  |  |
| Other | 21 (9.5%) | 21 (9.5%) |  |  |
| Shopping | 7 (3%) | 9 (5%) |  |  |
| Social network | 68 (32%) | 74 (35%) |  |  |
| **UADI-2** |  |  | W = 25455 | 0.531 |
| Mean (SD) | 57 (15) | 56 (16) |  |  |
| Median | 56 | 56 |  |  |
| Min - Max | 27 - 99 | 25 - 106 |  |  |

UADI-2: Internet Use-Abuse and Addiction (Questionario Uso-Abuso e Dipendenza da Internet 2)

• The authors should report basic multicollinearity checks (given conceptual overlap between PIU and PMPU) and, if feasible, include confidence intervals for key coefficients.

*We thank the reviewer for raising this important point. As the issue of multicollinearity overlaps with the sixth comment raised by Reviewer 1, we refer the reviewer to our response above, where the issue is addressed in detail. Regarding the comment on confidence intervals, we have now included 95% confidence intervals for autistic traits and K10 in the ANCOVAs results. All confidence intervals are reported in Supporting Information (S9 to S12).*

**Implications**
• The authors should more tightly connect intervention implications to the observed pattern (distress strongly linked to PIU/PMPU), highlighting distress-focused, CBT-informed coping/emotion-regulation strategies, with autism-adapted delivery when relevant.

*Thank you for the feedback. We have revised the future studies and conclusion sections to strengthen the potential implications of our findings for interventions (page 32-33).*

• The authors should consider specifying whether a fully de-identified dataset + codebook could be shared to support reproducibility while respecting ethical constraints.

*We have made the anonymized dataset and the corresponding codebook available on the OSF website (*[*https://osf.io/yvrfq/overview*](https://osf.io/yvrfq/overview)*).*

**Minor edits:** The authors should perform a brief language/typo pass (e.g., “cut-off”, “back button”, “imprisoned”).

*Thank you for noting this. The language and typos have been corrected throughout the manuscript.*
