# Supplementary material for: Association between Problematic Internet and Mobile Phone Use, autistic traits, and psychological distress among adults: A cross-sectional survey
Source: PLOS Ment Health. 2026 Jun 2;3(6):e0000524. doi: 10.1371/journal.pmen.0000524 (PMC13229353; doi:10.1371/journal.pmen.0000524)
Supplement: Attachment — Submitted filename: Response to Reviewers_2303.docx [file pmen.0000524.s015.docx]

**Authors’ Response to Reviewers’ Comments**

**Reviewer #1:**

The authors have responded constructively to all my comments, thank you. The changes are well implemented. The only remaining concern is about the AQ imagination subscale sensitivity analysis, which was performed but not included in the manuscript or supplementary materials. The authors offered to do it if required. I would leave the decision to the editor. Otherwise, the manuscript is for me publishable in this form.

*Thank you for your comments and feedback. We have now added the sensitivity analyses excluding the AQ Imagination subscale to the Supplementary Materials (S2).*

**Reviewer #2:**

The authors have addressed most of the reviewer’s major concerns and the revised manuscript is substantially improved, particularly in clarifying the definitions and measurement of PIU/PMPU, introducing the I-PACE framework earlier, strengthening the distinction between autistic traits and clinical ASD, and adding a sensitivity analysis for age. However, a few issues remain only partially resolved in the manuscript itself, including:

(i) clearer reporting of the IAT subsample,
(ii) fuller presentation of confidence intervals for key results,
(iii) a more explicit statement on data/code/codebook availability, and
(iv) a final language edit.

As a whole, the revision is largely responsive, and the remaining issues appear addressable without major additional analyses.

*Thank you for your comments. We have addressed each point in detail below:*

i. *The reference to the IAT subsample has been moved from the "Dependent variables” section to the “Survey” section (page 11, lines 2–6), where additional details have been provided. Supporting Information S1 reports the differences between participants who completed the IAT and those who did not.*
ii. *Confidence intervals have now added to the correlation analyses (Table 3 and Table 4), the description of Table 2 (see page 19, lines 13-17 and page 20, line 1), as well as to the MMR and MANCOVA results. The abstract has been updated reporting CIs, and the script in OSF has also been revised to include these new calculations.*
iii. *We have added a more detailed description of the files publicly available in the OSF repository to the “Data availability” statement in the paper.*
iv. *The remaining typos have been corrected.*
